# Palliative care in severe mental illnesses

**DOI:** 10.1186/s12904-023-01152-1

**Published:** 2023-03-30

**Authors:** Eva Katharina Masel, Bárbara Antunes, Christian Schulz-Quach

**Affiliations:** 1 Division of Palliative Medicine, Department of Medicine I, Medical University of Vienna, Waehringer Guertel 18-20, Vienna, 1090 Austria; 2 Primary Care Unit, Department of Public Health and Primary care, Palliative and End of Life Care Research Group, University of Cambridge, Cambridge, UK; 3 Centre for Mental Health, University Health Network, Toronto, Canada; 4 Division of Psychosocial Oncology, Department of Supportive Care, Princess Margaret Cancer Centre, University Health Network, Toronto, Canada; 5 Division of Consultation and Liaison Psychiatry, Department of Psychiatry, Temerty Faculty of Medicine, University of Toronto, Toronto, Canada

**Keywords:** Hospice and Palliative Care Nursing, Mental Health, Palliative Care, Psychiatry, Psychosocial Functioning

## Abstract

In this editorial, we highlight the interaction between patients who are diagnosed with severe mental illness and their treatment within palliative care, a clinical area of specialized focus which has a multitude of complex impacts on affected patients, their (chosen) family members and caregivers, as well as the healthcare professionals who are caring for them.

It cannot be denied that there is a stigma associated with both mental illness and palliative care. While palliative care is less often understood within the continuum of care it provides and is sometimes perceived as “the last resort when there is nothing more to be done”, mental health issues are often underdiagnosed, minimized or not treated with a sufficient degree of interprofessional collaboration. Indeed, universal access to palliative care and end-of-life care for patients suffering from serious mental illnesses remains an unmet goal [1].

Although most healthcare systems separate mental health from physical health services, creating systemic barriers to integrated palliative care for patients with severe mental illnesses, some medical fields, such as clinical psychiatry, are reversely providing care within a palliative care frame. Indeed, palliative psychiatry is an evolving field which focuses on mental illnesses that are severe, refractory, and often unresponsive to conventional psychiatric and psychosocial treatments.

Palliative psychiatry encompasses a wide range of issues, including widely-known mental health conditions, like anxiety or depression, treatment-refractory serious mental illnesses, neuropalliative care and symptom burden at various levels. Additionally, it addresses ethics and psychosocial problems, psychological distress, personhood, the wish and will to die, dignity, loneliness, social isolation, as well as psychopharmacology. Furthermore, the “3 Ds” of palliative psychiatry include depression, dementia, and delirium [2] and it is worth mentioning that psychiatric comorbidities are common in patients receiving palliative care.

While palliative care generally attempts to improve quality of life at any stage along the disease trajectory and to reduce symptom burden, palliative psychiatry focuses on mental health rather than physical issues [3]. However, quality of life is a broad concept which needs to be redefined in the face of severe mental illness. In order to provide a patient with the best care possible, mental health aspects should not be outsourced but be part of a comprehensive assessment [4].

This raises the question of whether the (repeated) failure of various therapy attempts could lead to a shift in therapy goals. This question is critical in palliative psychiatry, where therapy attempts may involve freedom constraints. Considering ethical implications, defining realistic therapy goals and weighing a benefit-harm ratio seem all essential elements, especially after numerous failed therapy [5].

This is where palliative care’s core competencies come into play, through the assessment of distressing circumstances and the development of an individual-focused interprofessional treatment plan. A palliative service certainly goes beyond pharmacology and it is essential to never forget the ABCDs of caring: **a**ttitude, **b**ehaviour, **c**ompassion and **d**ialogue [6]. In both psychiatry and palliative care, holistic approaches are paramount to alleviate symptoms, whether visible or invisible. However, new exciting pharmacological approaches to severe mental illness are also in place, such as psychedelics, psychedelic-associated psychotherapy [7, 8] and ketamine for suicidality [9].

We are now welcoming submissions to our Collection of articles titled “Palliative Care in Severe Mental Illnesses”. More details can be found here: https://www.biomedcentral.com/collections/PCSMI. We would like to invite you to contribute and illuminate the many points of contact between serious mental health issues and palliative care so that the bio-psycho-socio-spiritual model that constitutes comprehensive care can be made accessible and mapped. Barriers, contradictions, burning issues and deficits should also find a place, as we do not live in an ideal world [10].

We hope that this Collection will inspire you to recognize the versatility of palliative care. Cicely Saunders said, “Good care can reach the most hidden places”. This also means listening, asking, allowing for the full spectrum of human emotion to be safely experienced and addressed by all involved, educating oneself and giving mental health issues the necessary space they need.

## Data Availability

not applicable.
